# Role of insulin resistance and the gut microbiome on urine oxalate excretion in ob/ob mice

**DOI:** 10.14814/phy2.15357

**Published:** 2022-07-18

**Authors:** Hong Xiang, Haoqing Chen, Yuanyuan Liu, Dylan Dodd, Alan C. Pao

**Affiliations:** ^1^ Division of Nephrology, Department of Medicine Stanford University School of Medicine Palo Alto California USA; ^2^ Department of Pathology Stanford University School of Medicine Stanford California USA; ^3^ Department of Microbiology & Immunology Stanford University School of Medicine Stanford California USA; ^4^ Department of Urology Stanford University School of Medicine Palo Alto California USA; ^5^ Veterans Affairs Palo Alto Health Care System Palo Alto California USA

**Keywords:** leptin, microbiome, obesity, oxalate

## Abstract

Ob/ob mice have recently emerged as a model for obesity‐related hyperoxaluria as they are obese and excrete more urine oxalate compared to wild type mice. Ob/ob mice are deficient of leptin and develop obesity with hyperphagia and hyperinsulinemia. We hypothesized that insulin resistance and the gut microbiome contribute to hyperoxaluria in ob/ob mice. We developed a new liquid chromatography–mass spectrometry assay for urine oxalate and first compared urine oxalate excretion in ob/ob mice before and after ablation of intestinal bacteria with a standard antibiotic cocktail. We then compared urine oxalate excretion in ob/ob mice before and after leptin replacement or pioglitazone treatment, two maneuvers that reduce insulin resistance in ob/ob mice. Ob/ob mice excreted more oxalate into the urine in a 24‐h period compared to wild type mice, but antibiotic, leptin, or pioglitazone treatment did not change urine oxalate excretion in ob/ob mice. Unexpectedly, we found that when food intake was carefully matched between ob/ob and wild type mice, the amount of 24‐h urine oxalate excretion did not differ between the two mouse strains, suggesting that ob/ob mice excrete more urine oxalate because of hyperphagia. Since the level of urine oxalate excretion in wild type mice in our study was higher than those reported in prior studies, future work will be needed to standardize the measurement of urine oxalate and to define the range of urine oxalate excretion in wild type mice so that accurate and valid comparisons can be made between wild type mice and ob/ob mice or other mouse models.

## Graphical abstract

The purpose of this study was to test whether insulin resistance and/or the gut microbiome contributes to hyperoxaluria in ob/ob mice. Ob/ob mice excrete more oxalate into the urine in a 24‐hour period compared to wild type mice, but antibiotic, leptin, or pioglitazone treatment did not change urine oxalate excretion for either ob/ob or wild type mice. Unexpectedly, we found that when food intake was carefully matched between ob/ob and wild type mice, the amount of 24‐hour urine oxalate excretion did not differ between the two mouse strains, suggesting that ob/ob mice excrete more oxalate into the urine because of hyperphagia.

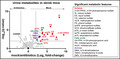

## 1 INTRODUCTION

Each year in the United States over 1 million patients are diagnosed with kidney stones (Litwin & Saigal, 2012). The prevalence of kidney stones has been estimated to be 11% for men and 7% for women and continues to rise, now twice as high as it was three decades ago (Scales, et al., 2012; Stamatelou et al., 2003). The increasing prevalence of kidney stones coincides with the increasing prevalence of obesity, insulin resistance, and diabetes (Cameron et al., 2006; Daudon et al., 2006; Eisner et al., 2010; Taylor et al., 2005a, 2005b; Weinberg et al., 2014; West et al., 2008). Persons with obesity, insulin resistance, or diabetes tend to excrete urine that favors the formation of calcium oxalate stones (Al‐Hayek et al., 2013; West et al., 2008) and uric acid stones (Maalouf et al., 2004). With the rise in prevalence of obesity, insulin resistance, and diabetes, there has also been a parallel rise in urine oxalate excretion (Sakhaee, 2018; Xu et al., 2017), a key risk factor for calcium oxalate kidney stones (Curhan & Taylor, 2008). It has been postulated that risk for kidney stones in obesity, insulin resistance, or diabetes can be partly explained by the tendency of these persons to develop hyperoxaluria, or high urine oxalate excretion (Kleinman, 2007; Lemann, et al., 1996; Taylor & Curhan, 2006, 2008).

Ob/ob mice have recently emerged as a model for obesity‐related hyperoxaluria as they are obese and excrete more urine oxalate compared to lean mice (Amin et al., 2018). Ob/ob mice are deficient of leptin and develop obesity with hyperphagia and hyperinsulinemia (Lindström, 2007). The proposed mechanisms underlying hyperoxaluria in ob/ob mice include the presence of intestinal/systemic inflammation, characterized by high levels of TNF, IFN‐γ, and IL‐6 in the jejunum and plasma (Amin et al., 2018). Direct application of these inflammatory cytokines to Caco‐2 BBe cells inhibits oxalate secretion, suggesting that inflammation can directly increase net intestinal oxalate absorption and urine oxalate excretion (Amin et al., 2018). There is also evidence that ob/ob mice have higher capacity for small intestinal paracellular oxalate absorption because they have reduced expression of tight junction proteins such as occludin, Zo‐1, claudin‐1 and 3 in the gastrointestinal tract (Bashir et al., 2019).

Two potential mechanisms underlying hyperoxaluria in ob/ob mice remain unaddressed. First, ob/ob mice are also a mouse model of insulin resistance, and insulin resistance—apart from obesity—could contribute to hyperoxaluria. Second, altered gut microbiota in ob/ob mice could also contribute to hyperoxaluria. Ob/ob mice have an altered microbiome (Ley et al., 2005) and a higher capacity for energy harvest from the diet (Turnbaugh et al., 2006). It is well known that certain members of the gut microbiota can increase urinary oxalate excretion in rodents and humans. *Oxalobacter formigenes* is an anaerobic bacterium that degrades oxalate in the gut (Siva et al., 2009) and stimulates intestinal oxalate secretion, ultimately limiting net intestinal absorption and urine oxalate excretion (Arvans et al., 2017). Several clinical studies have linked gut colonization with *O. formigenes* to a lower risk for stone recurrence in patients with calcium oxalate stones (Kaufman et al., 2008; Sidhu et al., 1999; Siener et al., 2013). Moreover, recent evidence in rodent models suggests that the microbial community in the gut, rather than individual bacterial species, is critical for influencing oxalate levels in the host (Miller et al., 2017).

We hypothesized that insulin resistance and/or the gut microbiome contributes to hyperoxaluria in ob/ob mice. We first compared urine excretion of oxalate and related metabolites in ob/ob mice before and after ablation of intestinal bacteria with a standard antibiotic cocktail. Using a newly developed liquid chromatography–mass spectrometry (LC–MS) assay for urine oxalate, we then compared urine oxalate excretion in ob/ob mice before and after leptin replacement or pioglitazone treatment, two maneuvers that reduce insulin resistance. Although we found that ob/ob mice excreted more urine oxalate compared to wild type mice, we observed no effect of antibiotics, leptin, or pioglitazone treatment on urine oxalate excretion in ob/ob mice. Unexpectedly, we found that when food intake was carefully matched between ob/ob and wild type mice, the amount of urine oxalate excretion did not differ between the two mouse strains.

## 2 MATERIALS AND METHODS

### 2.1 Animals

*Ob/ob* and wild type mice (males; 8–10 weeks old; on a C57BL/6J background) were obtained from The Jackson Laboratory and bred in maximum barrier rooms that were free of pathogens and parasites (RB06 & RB07). Adult age‐ and weight‐matched mice were used for experiments and housed individually in a non‐barrier room where the light/dark cycle was 12:12, lights on at 6:30 a.m. All mice were maintained with standard chow with an energy density of 3.1 kcal/g (Teklad Global 18% Protein Rodent Diet, Envigo) and ad libitum tap water. The 2918 Teklad diet does not contain supplemental oxalate but does contain oxalate because of grain‐based ingredients and has been previously measured at 12 μmol of oxalate per gram (Jiang et al., 2006). The oxalate content in 2918 Teklad diet indicates that mice ingested approximately 180 μmol of oxalate per day for the pair‐fed experiments (15 g diet/day) or 48 μmol of oxalate per day for the food‐restriction experiments (4 g diet/day).

### 2.2 Metabolic cage experiments

We acclimated mice to individual metabolic cages (Hatteras Instruments) for 5 days prior to urine collection. Between 8:30 and 9:00 a.m. every day, mice were weighed, placed in individual metabolic cages, and presented with gel food. Gel food (1% agarose solution with 0.52 ml water/kcal) was prepared as previously described (Nizar et al., 2018) and administered in an amount of 15 g per mouse per day. In separate food restriction experiments, we limited gel food intake to 4 g per mouse per day. Weights of mice in all experiments were stable by the last day of the acclimation period. Thereafter, urine was collected over 24 h under mineral oil, acidified with 4 N HCl (10 μl/200 μl urine), and stored in a −80°C freezer. We chose a 5‐day acclimation period because this is the time needed to reach steady‐state urine oxalate excretion in response to a change in dietary oxalate (Amin et al., 2018; Mitchell et al., 2019).

### 2.3 Metabolomic analysis

Twenty‐four‐hour urine samples from antibiotic and mock‐treated ob/ob mice (*n* = 6 per group) were sent to Metabolon for metabolomic analysis using a LC–MS platform as previously described (Evans et al., 2014; Guo et al., 2015). Approximately 774 compounds of known identity were detected. Original scaled peak areas were log_2_‐transformed and analyzed for statistical significance using false discovery rate correction via the two‐stage step‐up method of Benjamini, Krieger, and Yekutieli as implemented in GraphPad Prism 9 (GraphPad Software, Inc.). The criterion for biological significance was a 4‐fold change and for statistical significance was a *q* < 0.05.

### 2.4 Measurement of urine oxalate excretion by stable isotope dilution LC–MS

Urine samples (20 μl) were combined with an acidified internal standard (IS) solution (10 μl; [^13^C_2_]‐oxalate, 0.5 mM in 5% TFA), and protein was precipitated by addition of organic solvent (90 μl; ACN/MeOH 3:1 mixture). After centrifugation, sample supernatants (20 μl) were diluted with 0.1% TFA (80 μl), and samples were analyzed by LC–MS using an Agilent 1290 Infinity II UPLC and detected using an Agilent 6545XT Q‐TOF equipped with a dual jet stream electrospray ionization (ESI) source operating under extended dynamic range (EDR 1700 m/z). Eluent A consisted of 0.1% formic acid (v/v) in water, and eluent B consisted of 0.1% acetic acid (v/v) in 100% acetonitrile. Samples (5 μl) were injected via refrigerated autosampler into mobile phase, and separation at 50°C was achieved with a Thermo Acclaim OA column (3 μm, 2.1 × 150 mm) equipped with a 2.1 mm I.D. filter cartridge, 0.2 μm (Thermo). Two pumps were used, one for analytical separation, and the second to introduce organic solvent via a post‐column Tee to increase ESI signal intensity and stability. Pump A delivered solvent to the analytical column at a flow rate of 0.2 ml/min with the following gradient: 0–3 min, 0% B, 3–3.5 min, 0%–100% B, 3.5–4.5 min, 100% B, 4.5–4.7 min, 100%–0% B, 4.7–6.3 min, 0% B. Pump B delivers 100% B at 0.2 ml/min to the post‐column Tee. Detection was conducted in ESI negative mode. ESI parameters included a gas temperature of 150°C, drying gas flow of 6 L/min, nebulizer pressure of 30 psi, sheath gas temperature of 150°C and sheath gas flow of 11 L/min, capillary voltage of 4000 V, and fragmentor voltage of 140 V. Peak areas for oxalate ([M‐H]‐, m/z 88.9880) were normalized using the IS (oxalate‐^13^C2; [M‐H]‐, m/z 90.9947), and concentrations were determined by comparison to calibration curves prepared from dilution series of authentic standard spiked in water as a surrogate matrix.

### 2.5 Validation and comparison of LC–MS and colorimetric assays for oxalate determination

We used LC–MS and a modified version of the colorimetric oxalate oxidase assay kit from Trinity Biotech (Cat. # 591‐D) for validation and comparison of oxalate measurement. The colorimetric kit is designed for measuring oxalate in human urine in which large volumes of urine are routinely available. To adapt the colorimetric kit for measuring oxalate in smaller volumes of mouse urine, we proportionally decreased all reagents in the assay kit by 200‐fold. Twenty microliters of sample were diluted with 25 μl of sample diluent. The pH was checked using a pH strip, then adjusted to the range of 5.0–9.0 by addition of 1 M NaOH. Twenty microliters of diluted (pH adjusted) samples were added to a tube containing 3.65 ± 0.1 mg of activated charcoal, mixed intermittently for 5 min, and centrifuged at 1500*g* for 5 min. The supernatant was transferred to a fresh tube and centrifuged again at 15,000*g* for 1 min. Five microliters of supernatant was then mixed with 100 μl of Oxalate Reagent A and 10 μl of Oxalate Reagent B in wells of a flat bottom microplate (Corning Life Sciences). After incubation for 5 min at room temperature, the absorbance at 590 nm was recorded using a Synergy H1M multimode plate reader (Biotek). Concentrations were determined by comparison to a calibration curve generated with sodium oxalate.

For comparison of LC–MS and colorimetric assays for oxalate determination, we used certified reference material traceable to NIST (SRM 8040) from Trinity Biotech as reference standards. These included lyophilized human urine with normal oxalate (Cat. # O6627) and elevated oxalate (Cat. # O6502). To assess intra‐run and inter‐run precision of LC–MS and colorimetric assays, we analyzed oxalate levels in these two reference standards in triplicate on three separate days with both assays. To compare differences between LC–MS and colorimetric assays, we analyzed 18 different mouse urine samples from our study with both assays and generated a Bland–Altman plot with GraphPad Prism 9.

### 2.6 Measurement of urine creatinine

We mixed urine samples or calibration standards with creatinine‐D3 as an internal standard (IS) in a V‐bottom, polypropylene 96‐well plate, and then extracted by mixing with an extraction solution (75% acetonitrile/25% methanol at 1:3 ratio). The plate was covered with a lid and centrifuged at 5000*g* for 15 min at 4°C. The supernatant was transferred to a V‐bottom polypropylene plate, sealed, and then subjected to LC–MS analysis. Samples were injected via refrigerated autosampler into mobile phase, chromatographically separated by an Agilent 1290 Infinity II UPLC, and detected using an Agilent 6545XT Q‐TOF equipped with a dual jet stream ESI source operating under EDR (1700 m/z) in positive ionization mode. Table S2 provides chromatography and instrument conditions. MS1 spectra were collected in centroid mode, and peak assignments in samples were made based on comparisons of retention times and accurate masses from authentic standards using MassHunter Quantitative Analysis v.10.0 software from Agilent Technologies. We quantified creatinine concentration in urine samples from calibration curves constructed with an authentic creatinine standard using isotope‐dilution mass spectrometry with creatinine‐D3 as an IS.

### 2.7 Measurement of urine glucose

We used a dye‐based glucose assay kit from Abcam (Cat. # ab272532) for the determination of urine glucose. Thirty microliters of calibration standards or appropriately diluted urine samples were combined with 300 μl of assay reagent in 1.5 ml Eppendorf tubes and heated at 100°C for 8 min in a thermomixer. Samples were then transferred to wet ice and cooled for 4 min, then 100 μl (in duplicate) was transferred to a clear flat‐bottom 96‐well plate, and the absorbance at 630 nm was recorded using a Synergy H1M multimode plate reader from Biotek. We determined glucose concentrations in urine samples by comparison to a calibration curve with glucose as a standard.

### 2.8 Intestinal bacteria ablation

Ob/ob and wild‐type C57BL/6J mice were given an antibiotic cocktail in the drinking water for 5 days. The antibiotic cocktail comprised of ampicillin (1 g/L, Sigma), neomycin (1 g/L, Fisher), metronidazole (0.5 g/L, Fisher), and vancomycin 1 g/L, Fisher). This antibiotic cocktail regimen has been used to deplete and ablate intestinal commensal bacteria in mice (Ivanov et al., 2008).

### 2.9 Leptin replacement

Lyophilized leptin (Sigma, catalog #L3772) was diluted in sterile Tris HCl, pH 8.0 to make a leptin stock solution of 1 mg/ml. Next, the stock solution was diluted in 0.9% saline to make a leptin working solution of 0.125 mg/ml. Leptin was administered at a dose of 0.1 mg/kg body weight twice daily through an intra‐peritoneal injection volume of 200 μl. Leptin was administered at 8:30 a.m. and 8:30 p.m. each day for a total of 18 days. This dosing regimen for leptin normalizes circulating concentration and adipose tissue expression of adiponectin and obesity‐associated oxidative stress and inflammation in ob/ob mice (Frühbeck et al., 2017). For mock replacement controls, 0.9% saline was administered to mice in a volume of 200 μl through intra‐peritoneal injection twice daily for 18 days.

### 2.10 Pioglitazone treatment

Pioglitazone (Sigma, catalog #E6910) was dissolved in 0.25% w/v carboxymethylcellulose and administered to mice via oral gavage at 30 mg/kg daily, administered at 8:30 a.m., for 14 days. This dosing regimen for pioglitazone improves insulin resistance, diabetes, and raises serum adiponectin levels in ob/ob mice (Kubota et al., 2006). For mock treatment controls, an equivalent volume of 0.25% w/v carboxymethylcellulose solution relative to the treatment group was administered to mice via oral gavage daily for 14 days.

### 2.11 Statistical analysis and data presentation

Data are reported as mean ± *SE*. Statistical comparisons were made by 2‐way ANOVA with Tukey correction for multiple comparisons using GraphPad Prism 9. The criterion for significance was *p* < 0.05.

## 3 RESULTS

### 3.1 Effect of ablation of intestinal bacteria on urine metabolome in ob/ob mice

**FIGURE 1:**
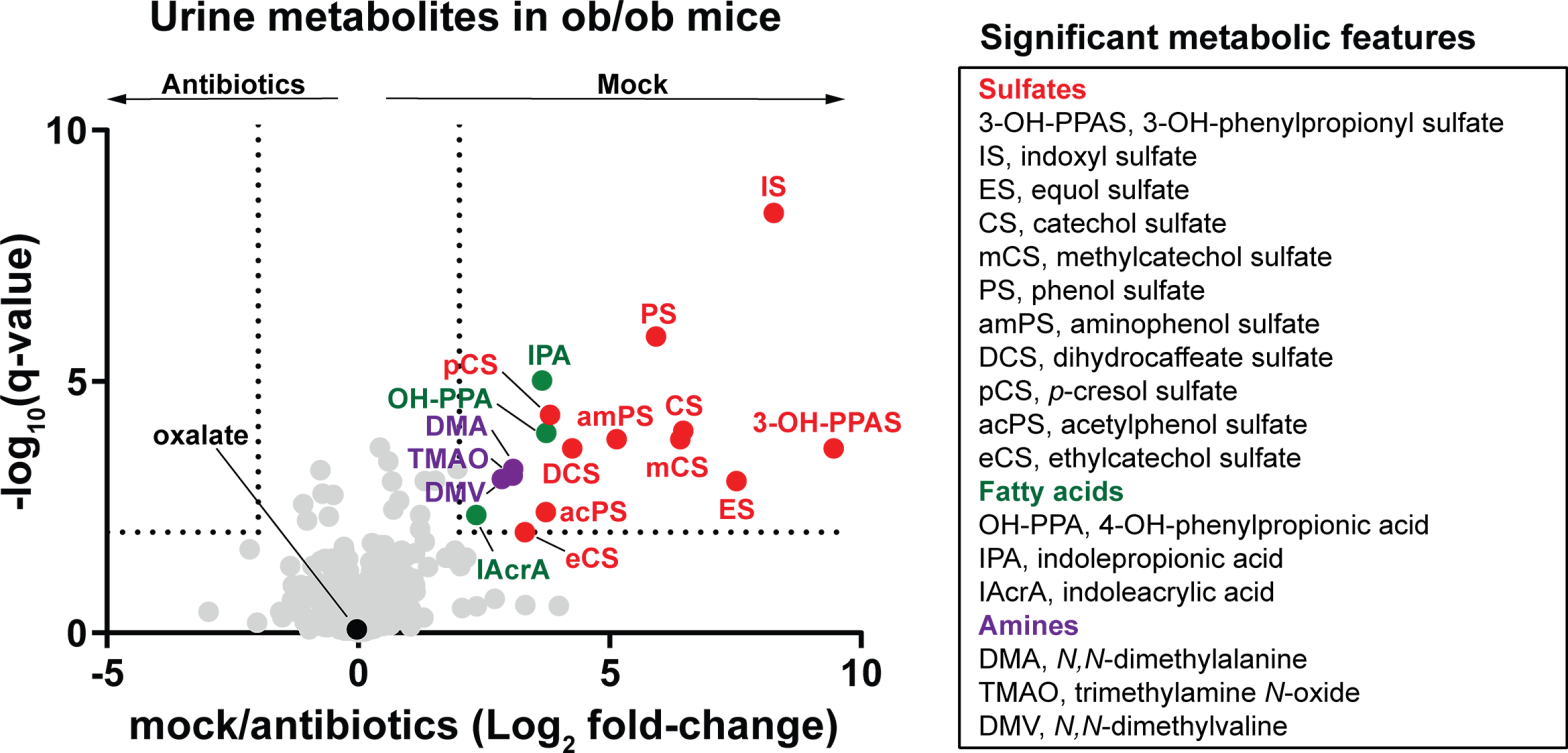
Effect of ablation of intestinal bacteria on urine metabolome in ob/ob mice. Twenty‐four‐hour urine samples were sent to Metabolon, Inc. for metabolomic analysis using a liquid chromatography–mass spectrometry platform. A volcano plot is shown with differentially abundant named metabolites (|fold change|>4, *q* < 0.05) are listed. *n* = 6 per group.

To evaluate the contribution of the gut microbiome to urine metabolite excretion in ob/ob mice, we treated ob/ob mice with an antibiotic cocktail for 5 days to ablate intestinal bacteria and compared the 24‐h urine metabolomes between mock and antibiotic treated mice (Table S2). Antibiotic treatment reduced urine excretion of 17 metabolites (|fold change|>4, *q* < 0.05; Figure 1). These metabolites included (i) sulfated aromatic metabolites; (ii) aromatic fatty acids; and (iii) amine containing compounds. Most of these metabolites are known to arise from or be affected by microbial metabolism in the gut (Van Treuren & Dodd, 2020), suggesting that antibiotic treatment successfully ablated the gut microbiota. Notably, none of these metabolites relate to oxalate metabolism, and in these data from shotgun metabolomics, the urine oxalate levels were not significantly different between mock and antibiotic treated ob/ob mice (Figure 1).

### 3.2 Development of a stable isotope dilution LC–MS assay for urine oxalate

LC–MS assays have superior characteristics over alternative methodologies for quantifying urine oxalate such as colorimetric coupled enzyme assays, which may be subject to sample interference (Salido et al., 2006), and ion chromatography (IC‐HPLC), which requires specialized instrumentation. However, current LC–MS methods require chromatography with long regeneration times or time‐consuming pre‐analytical sample preparation, such as sample dry down or solid phase extraction steps. Here we sought to develop a rapid and robust LC–MS assay that could be used on most modern systems. Initially, we scouted several different methods using either hydrophilic interaction liquid chromatography or reversed‐phase chromatography using either a C18 column, a porous graphitic carbon column, or an organic acid column. We chose a Thermo Acclaim™ organic acid column (with filter cartridge) because it provided the best peak shape, column retention, and signal sensitivity. We optimized the chromatographic method to improve peak shape and signal sensitivity in the following ways: (i) Use of 0.1% formic acid in aqueous solvent to maintain low pH and proper peak shape; (ii) addition of 0.1% acetic acid to organic solvent to enhance ionization; and (iii) introduction of additional organic solvent during elution via a post‐column Tee to further boost signal.

**FIGURE 2:**
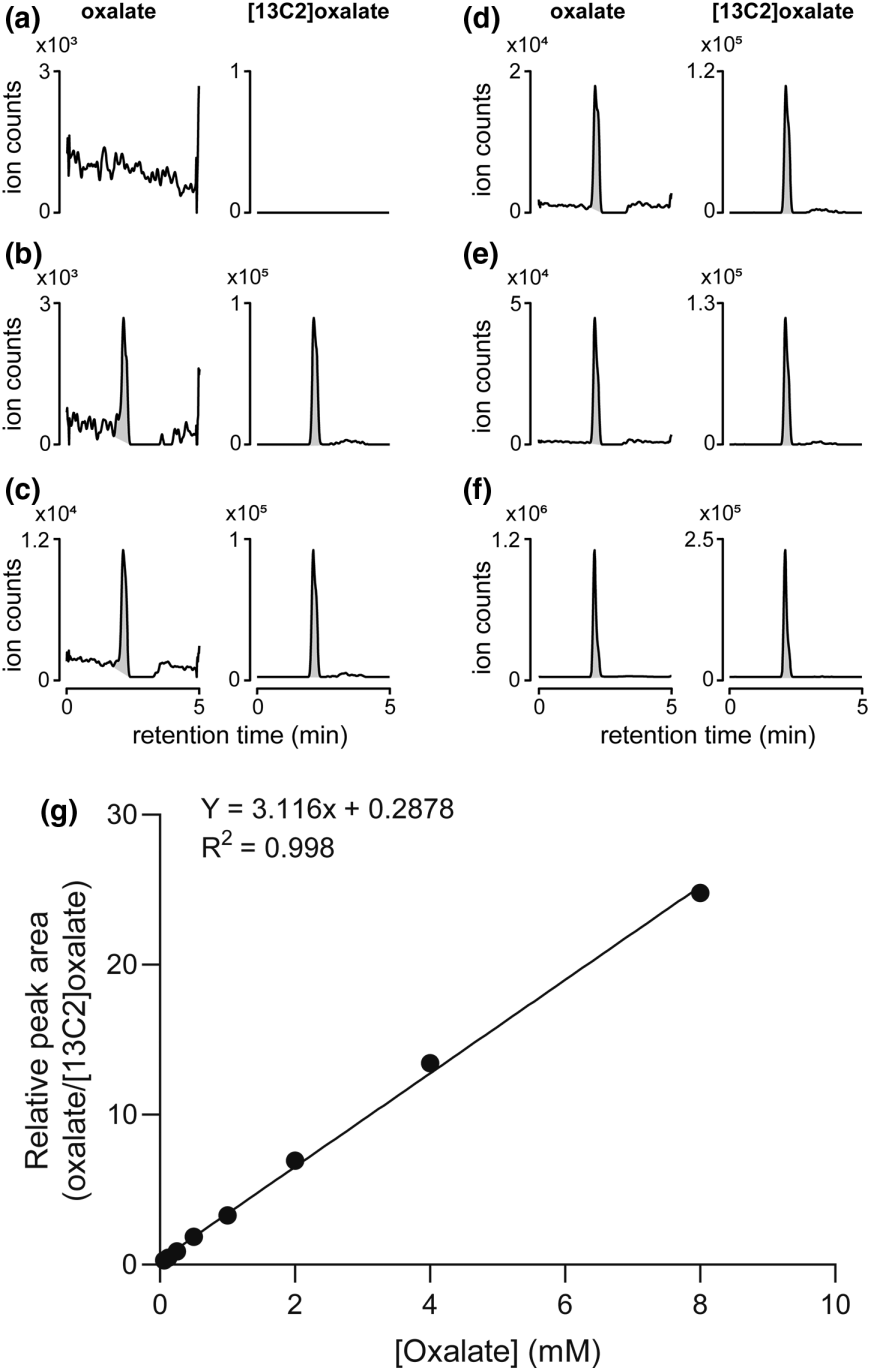
Extracted ion chromatograms of oxalate and [^13^C2]‐oxalate internal standard (IS) and calibration curve. (a) Double blank of pure water. (b) Blank water spiked with IS and processed through the sample preparation steps. (c–f) Water spiked with 0.013, 0.033, 0.082, 1.28 mM oxalate and IS and processed through the sample preparation steps. (g) Calibration curve generated with oxalate diluted from 8 mM to 62.5 μM in water and processed through sample preparation steps. Peak areas for oxalate were divided by the peak areas for the internal standard and plotted against the concentration of oxalate in the calibration samples.

The representative chromatograms of a pure water blank, the processed sample of water blank spiked with IS ([^13^C2]‐oxalate), and processed samples of water spiked with oxalate and IS, are shown in Figure 2. The pure water blank showed only background noise (Figure 2a), while the blank that was processed through the sample preparation step resulted in an oxalate peak above the LOD of the method (Figure 2b). Figure 2c–f show the water samples spiked with 0.013, 0.033, 0.082, 1.28 mM oxalate, and IS and processed through the sample preparation steps. The peaks are sharp and symmetric, with a retention time of 2.12 min and peak width of 0.15 min. An eight‐point calibration curve prepared in water ranging from 62.5 μM to 8 mM displayed excellent linearity with an *R* ^2^ of 0.998 (Figure 2g).

### 3.3 Validation and comparison of LC–MS and colorimetric assays for oxalate determination

**FIGURE 3:**
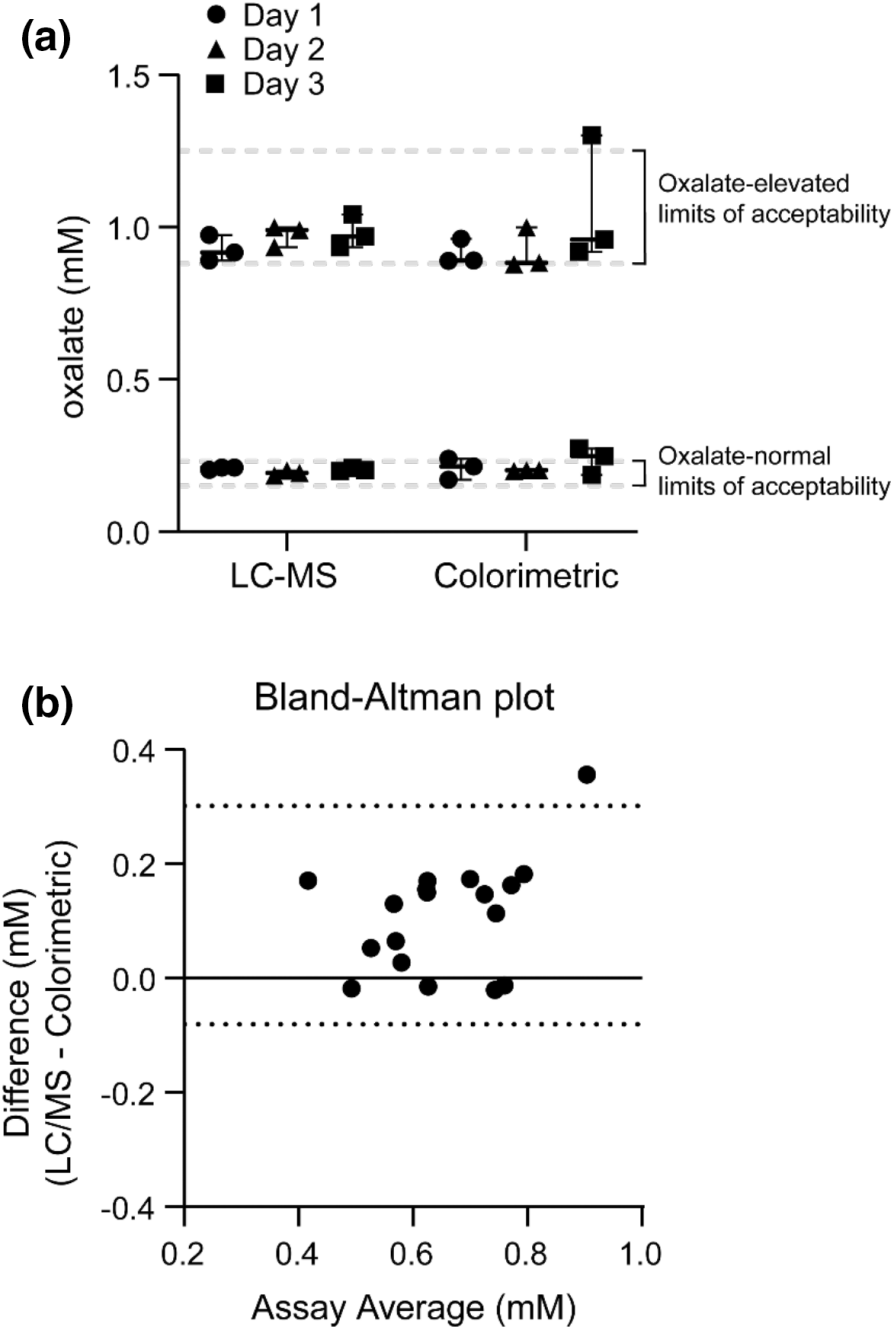
(a) Accuracy of LC–MS and colorimetric assays in measurement of oxalate in human urine. Oxalate was assayed by certified reference material (normal and elevated) in triplicate on three separate days. Dashed lines show the limits of acceptability defined by the supplier. Box plots show the mean and range of oxalate levels. (b) Differences in mouse urine oxalate values between LC–MS and colorimetric assays. Eighteen randomly selected 24‐h urine samples from mice. Oxalate concentrations were measured by LC–MS and colorimetric assays. A Bland–Altman plot was generated using GraphPad Prism 9. Dashed lines show the 95% limits of agreement. There was a positive bias of 0.11 ± 0.097 in the LC–MS method relative to the colorimetric method. LC–MS, liquid chromatography—mass spectrometry.

To further evaluate the accuracy, intra‐run precision, and inter‐run precision of our LC–MS assay, we compared oxalate concentration measurements between our LC–MS assay and the colorimetric oxalate oxidase assay. We obtained samples that were certified for reference material for normal or elevated levels of oxalate in human urine (Trinity Biotech). Our LC–MS assay performed well: oxalate concentrations fell within the limits of acceptability defined by the supplier (Figure 3a). Intra‐assay variation was very low, showing an average % coefficient of variation (CV) of 2.9% for the normal urine oxalate controls and 4.6% for the elevated urine oxalate controls. Inter‐assay variation was also very low, with average % CV of 4.2% for the normal urine oxalate controls and 5.2% for the elevated urine oxalate controls. The performance of the colorimetric assay was also acceptable on average, but it showed more variation compared to the LC–MS assay (Figure 3a). Intra‐assay variation was 12.2% for the normal urine oxalate controls and 10.6% for the elevated urine oxalate controls. Inter‐assay variation was 13.8% for the normal urine oxalate controls and 9.4% for the elevated urine oxalate controls.

We then compared the two assays by measuring oxalate concentrations in 18 randomly selected mouse urine samples from our study. We used a Bland–Altman plot to compare differences between assays and found that our LC–MS assay showed a positive bias of 0.11 ± 0.097 mM compared to the colorimetric assay (Figure 3b). Based on the high precision of the LC–MS assay, we used this assay for the measurement of oxalate concentration in mouse urine samples for this study.

### 3.4 Effect of ablation of intestinal bacteria on urine oxalate excretion in ob/ob mice

**FIGURE 4:**
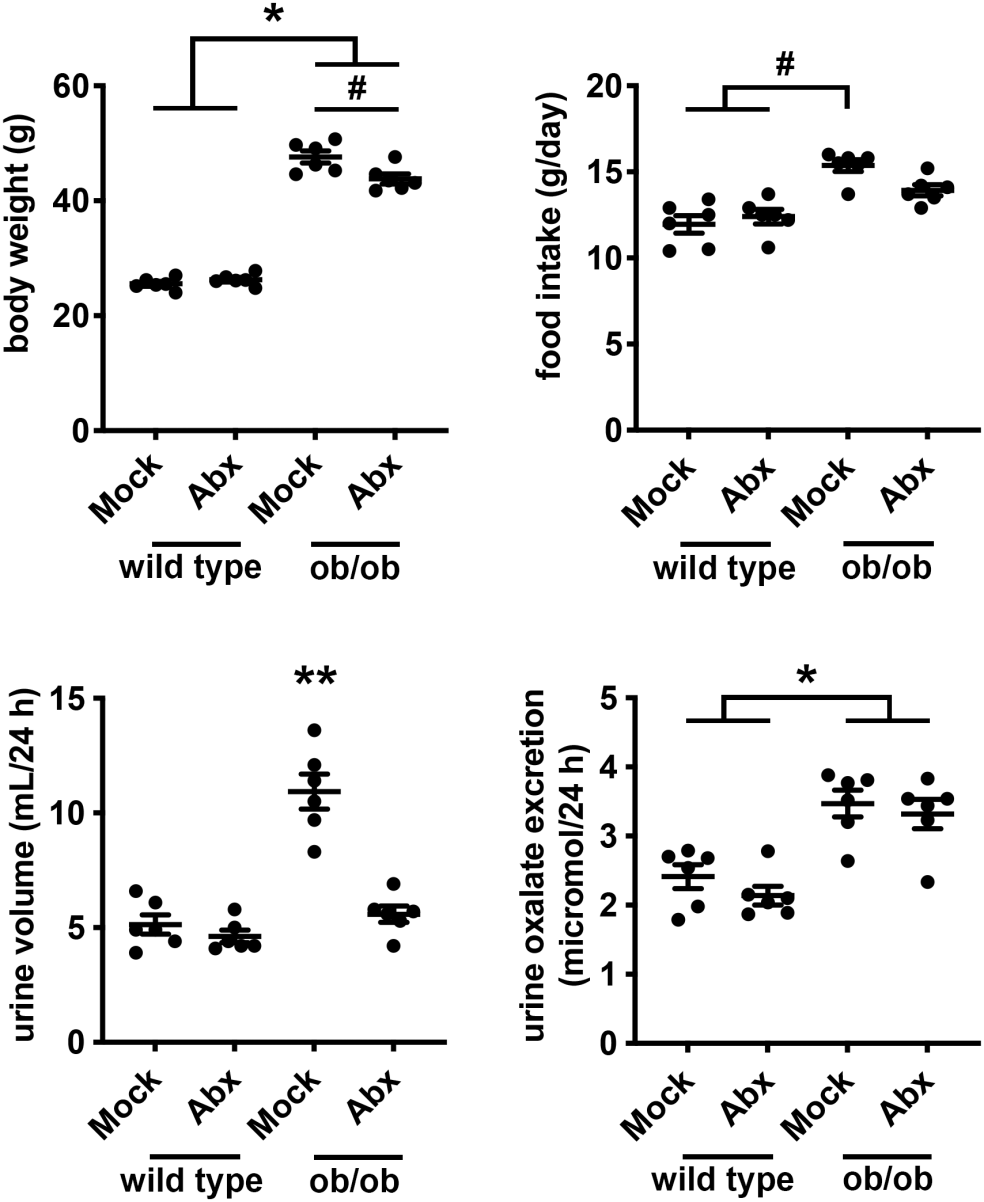
Daily body weight, food intake, urine volume, and urine oxalate excretion in wild type and ob/ob mice with mock or antibiotic (Abx) treatment. Wild type and ob/ob mice were given an antibiotic cocktail (ampicillin, neomycin, metronidazole, and vancomycin) in the drinking water for 5 days to ablate intestinal bacteria. Mock treatment contained no antibiotics in the drinking water. Mice were pair‐fed with gel food (1% agarose solution with 0.52 ml water/kcal) containing 15 g of chow per mouse per day and housed in individual metabolic cages. *n* = 6 per group. *p < 0.05 by 2‐way ANOVA with Tukey correction for the effect of genotype. ^#^ *p* < 0.05 by 2‐way ANOVA with Tukey correction for multiple comparisons. **p < 0.05 by 2‐way ANOVA with Tukey correction compared to other groups (wild type mice treated with mock or antibiotics, and ob/ob mice treated with antibiotics).

To test whether differences in gut microbiota can account for hyperoxaluria in ob/ob mice, we compared urine oxalate excretion between ob/ob and wild type mice after 5 days of mock or antibiotic treatment. Wild type mice treated with antibiotics demonstrated no differences in body weight or food intake to those with mock treatment (Figure 4). Ob/ob mice, as expected, weighed more than wild type mice after mock treatment (ob/ob 47.62 ± 1.04 g vs. wild type 25.55 ± 0.41 g, *p* < 0.05); antibiotic treatment decreased body weight of ob/ob mice after 5 days (−3.82 g, 95% confidence interval −0.58 to −7.06 g, *p* < 0.05; Figure 4). Twenty‐four‐hour urine volume was greater in ob/ob mice compared with wild type mice, and this greater urine volume was associated with glycosuria. Glycosuria is a known consequence of hyperglycemia in ob/ob mice (Erickson et al., 1996), and we observed glycosuria only in untreated ob/ob mice (Figure S1). Twenty‐four‐hour urine oxalate excretion in ob/ob mice was higher than wild type mice after mock treatment (ob/ob 3.47 ± 0.19 μmol vs. wild type 2.41 ± 0.17 μmol, *p* < 0.05), but the level of urine oxalate excretion did not change after 5 days of antibiotic treatment in either ob/ob or wild type mice (Figure 4).

### 3.5 Effect of leptin replacement and pioglitazone treatment on urine oxalate excretion in ob/ob mice

To test whether differences in insulin sensitivity can account for hyperoxaluria in ob/ob mice, we used two approaches. First, we administered leptin to determine whether leptin signaling can restore normal oxalate homeostasis in ob/ob mice and reduce urine oxalate excretion to a normal level. When ob/ob mice are administered exogenous leptin, it lowers body weight and improves markers of insulin resistance and systemic inflammation (Frühbeck et al., 2017). Second, we used a thiazolidinedione to test whether improving insulin sensitivity in ob/ob mice will lower urine oxalate excretion. A recent study showed that pioglitazone treatment of ob/ob mice improves insulin resistance and diabetes by increasing serum adiponectin levels and decreasing hepatic glucose production (Kubota et al., 2006).

**FIGURE 5:**
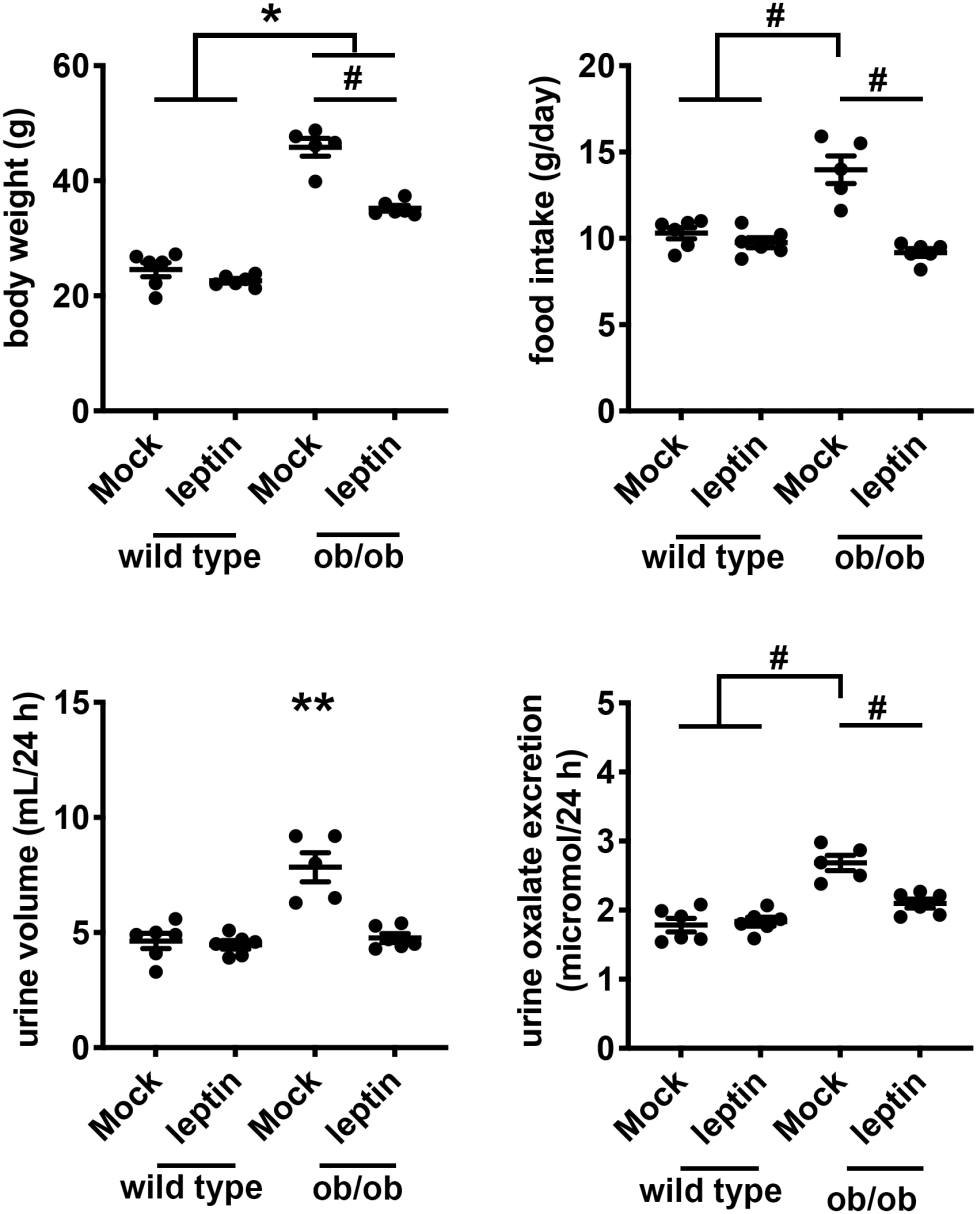
Daily body weight, food intake, urine volume, and urine oxalate excretion in wild type and ob/ob mice with mock or leptin replacement. Wild type and ob/ob mice were administered mock vehicle (0.9% saline twice daily by intraperitoneal injection) or leptin (0.1 mg/kg twice daily by intraperitoneal injection) for 18 days. Mice were pair‐fed and housed in individual metabolic cages so that chow intake was equivalent. *n* = 5 or 6 per group. *p < 0.05 by 2‐way ANOVA with Tukey correction for the effect of genotype. ^#^ *p* < 0.05 by 2‐way ANOVA with Tukey correction for multiple comparisons. **p < 0.05 by 2‐way ANOVA with Tukey correction compared to other groups (wild type mice treated with mock or leptin, and ob/ob mice treated with leptin).

Administration of leptin to ob/ob mice significantly decreased body weight (−10.92 g, 95% confidence interval −6.91 to −14.92 g, *p* < 0.05) by reducing food intake (−4.79 g, 95% confidence interval −2.92 to −6.66 g, *p* < 0.05), whereas administration of leptin to wild type mice did not affect body weight or food intake (Figure 5). Twenty‐four‐hour urine oxalate excretion was higher in ob/ob mice compared to wild type mice after mock replacement (ob/ob 2.68 ± 0.11 μmol vs. wild type 1.78 ± 0.10 μmol, *p* < 0.05), and the higher level of daily urine oxalate excretion was associated with higher level of daily food intake in ob/ob mice compared to wild type mice (ob/ob 13.98 ± 0.80 g vs. wild type 10.30 ± 0.33 g, *p* < 0.05; Figure 5). Leptin replacement lowered 24‐h urine oxalate excretion in ob/ob mice (−0.73 μmol, 95% confidence interval −0.32 to −1.15 μmol, *p* < 0.05) but did not change urine oxalate excretion in wild type mice.

**FIGURE 6:**
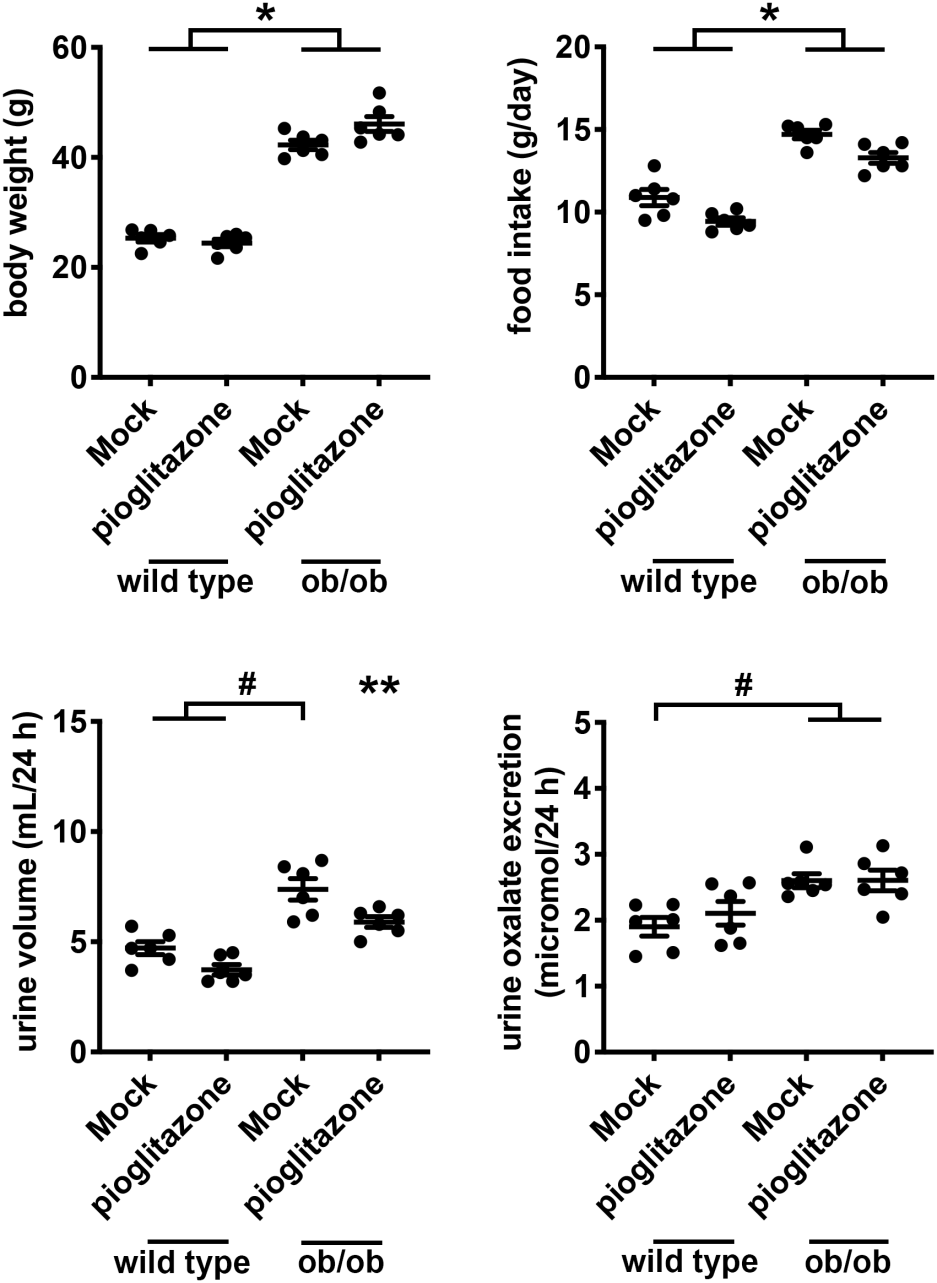
Daily body weight, food intake, urine volume, and urine oxalate excretion in wild type and ob/ob mice with mock or pioglitazone treatment. Wild type and ob/ob mice were administered mock vehicle (0.25% carboxymethylcellulose) or pioglitazone (30 mg/kg) via oral gavage for 14 days. Mice were pair‐fed and housed in individual metabolic cages so that chow intake was equivalent. *n* = 6 per group. *p < 0.05 by 2‐way ANOVA with Tukey correction for the effect of genotype. ^#^ *p* < 0.05 by 2‐way ANOVA with Tukey correction for multiple comparisons. **p < 0.05 by 2‐way ANOVA with Tukey correction between ob/ob mice and wild type mice treated with pioglitazone.

Pioglitazone treatment did not change body weight or food intake in either wild type or ob/ob mice (Figure 6). Twenty‐four‐hour urine oxalate excretion was higher in ob/ob mice after mock treatment (ob/ob 2.60 ± 0.11 μmol vs. wild type 1.90 ± 0.14 μmol, *p* < 0.05), and the higher level of daily urine oxalate excretion was associated with higher level of daily food intake in ob/ob mice (ob/ob 14.70 ± 0.26 g vs. wild type 10.88 ± 0.48 g, *p* < 0.05; Figure 6). Pioglitazone administration did not affect urine oxalate excretion in wild type or ob/ob mice.

### 3.6 Effect of leptin replacement and pioglitazone treatment on urine oxalate excretion in ob/ob and wild type mice under food restriction

**FIGURE 7:**
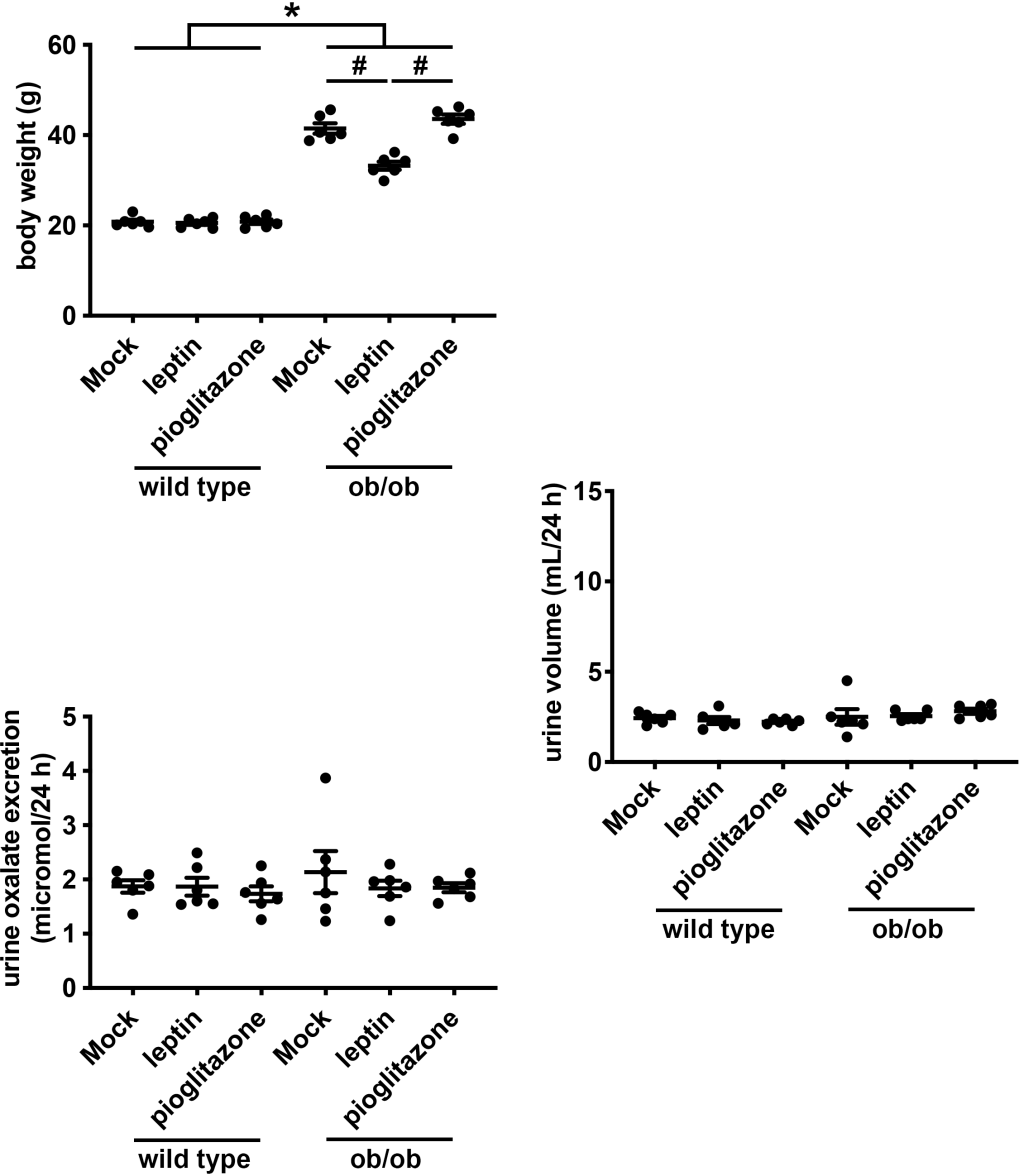
Daily body weight, urine volume, and urine oxalate excretion in wild type and ob/ob mice under food restriction in combination with mock replacement, leptin replacement or pioglitazone treatment. Mice underwent mock replacement (0.9% saline injection twice daily) for 18 days, leptin replacement for 18 days, or pioglitazone treatment for 14 days. Daily food intake of mice was limited to 4 g per mouse per day. *n* = 6 per group. *p < 0.05 by 2‐way ANOVA with Tukey correction for the effect of genotype. ^#^ *p* < 0.05 by 2‐way ANOVA with Tukey correction for multiple comparisons.

When ob/ob and wild type mice were pair fed with gel diets weighing 15 g per mouse per day, ob/ob mice consistently ingested the entire daily food volume whereas wild type mice did not. The net effect was that ob/ob mice as a group consumed more food than wild type mice despite our attempt to pair feed both groups of mice with the same quantity of chow. Since leptin replacement decreased daily food intake of ob/ob mice, and since this reduction in daily food intake correlated with a decrease in urine oxalate excretion (Figure 5), we surmised that leptin replacement decreases urine oxalate excretion in ob/ob mice by reducing their food intake. We thus tested whether leptin replacement ob/ob mice could still reduce urine oxalate when they are forced to ingest an identical amount of food as wild type mice. To ensure that daily food intake between ob/ob and wild type mice would be the same, we limited food intake to 4 g per mouse per day and re‐tested the effect of leptin replacement (and pioglitazone treatment) on urine oxalate excretion in ob/ob mice. With food restriction, ob/ob mice still weighed more than wild type mice (ob/ob 41.47 ± 1.15 g vs. wild type 20.80 ± 0.48 g, *p* < 0.05); ob/ob mice still lost weight with administration of leptin (−8.23 g, 95% confidence interval −4.73 to −11.74 g, *p* < 0.05) but not with treatment with pioglitazone (−2.08 g, 95% confidence interval −5.58 to +1.42 g, *p* = 0.46; Figure 7). Twenty‐four‐hours urine oxalate excretion was similar between ob/ob and wild type mice under conditions of food restriction; administration of leptin or pioglitazone did not affect urine oxalate excretion in either ob/ob or wild type mice (Figure 7).

### 3.7 Urine creatinine measurement in wild type and ob/ob mice

**FIGURE 8:**
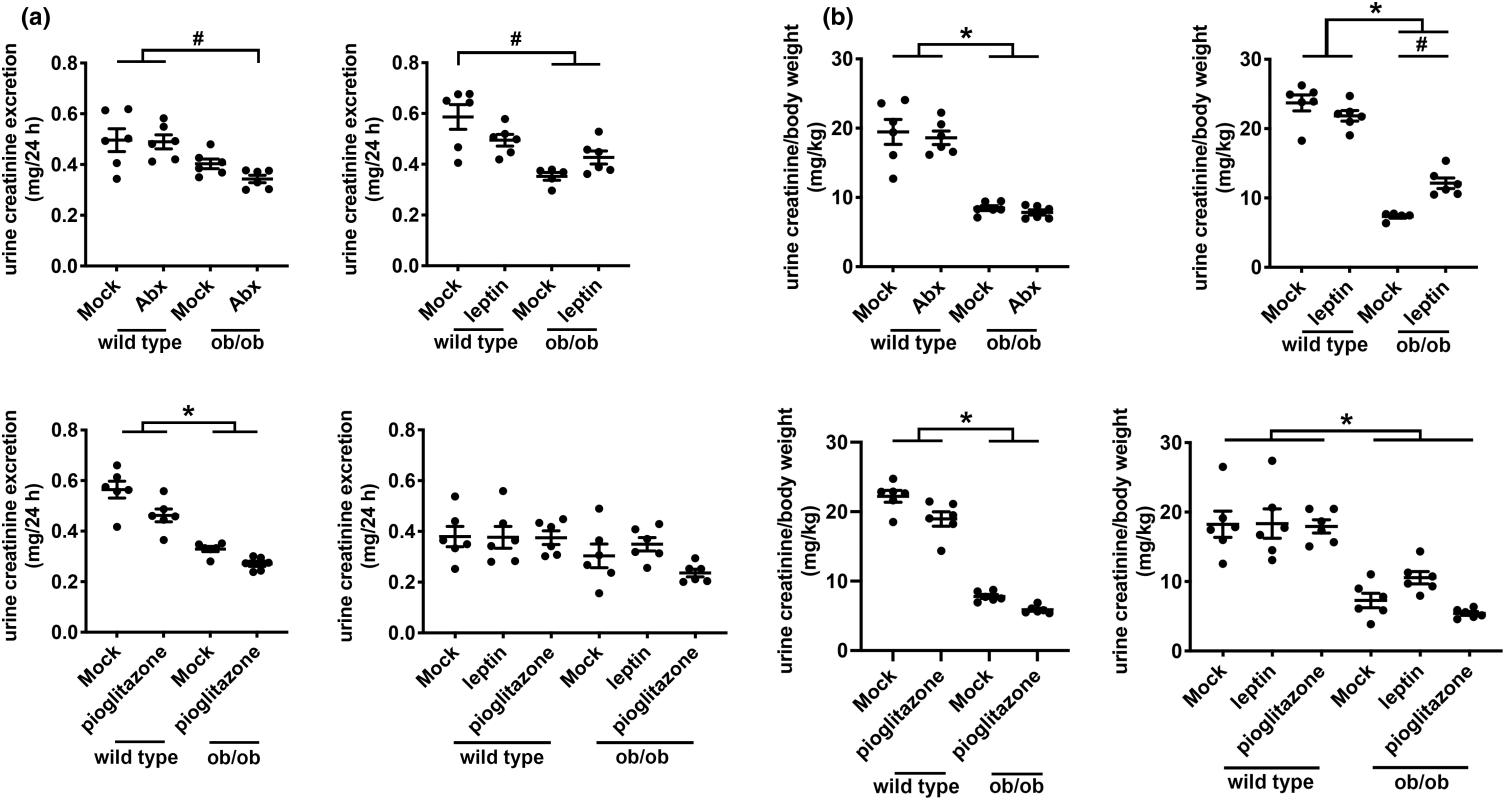
Urine creatinine excretion in wild type and ob/ob mice across different experiments. (a) Twenty‐four‐hour urine creatinine excretion between wild type and ob/ob mice in experiments with antibiotic treatment, leptin replacement, pioglitazone treatment, and food restriction. (b) Twenty‐four‐hour urine excretion normalized to body weight between wild type and ob/ob mice in experiments with antibiotic treatment, leptin replacement, pioglitazone treatment, and food restriction. ^#^ *p* < 0.05 by 2‐way ANOVA with Tukey correction for multiple comparisons. *p < 0.05 by 2‐way ANOVA with Tukey correction for the effect of genotype.

We measured creatinine concentration in 24‐h urine samples from mice for all experiments and found that ob/ob mice consistently excrete less creatinine into the urine compared to wild type mice when they were maintained at 15 g per mouse per day (Figure 8a). Differences in 24‐h‐urine excretion of creatinine between ob/ob and wild type mice dissipated when both mouse strains were maintained at 4 g per mouse per day (Figure 8a). When we normalized 24‐h urine creatinine excretion to body weight to yield a urine creatinine index for each mouse, we found that ob/ob mice excreted less urine creatinine (per body weight) compared to wild type mice regardless of the diet regimen they ingested (Figure 8b). The lower urine creatinine index for ob/ob mice indicates that they have lower lean body mass compared to wild type mice.

## 4 DISCUSSION

We used the ob/ob mouse model to test whether insulin resistance and/or the gut microbiome contributes to hyperoxaluria in obesity. First, we found that ablation of intestinal bacteria with an antibiotic cocktail did not affect urine excretion of oxalate or related metabolites in ob/ob mice. Second, we developed a rapid and robust LC–MS assay for measuring oxalate in mouse urine. Third, we used this LC–MS assay to determine that leptin deficiency and insulin resistance do not contribute to hyperoxaluria in ob/ob mice because leptin replacement or pioglitazone treatment did not affect urine oxalate excretion. Finally, we found that when ob/ob and wild type mice were carefully matched to ingest identical amounts of food, ob/ob mice excreted the same amount of oxalate into the urine as wild type mice. We conclude that ob/ob mice excrete more oxalate into the urine because of hyperphagia. Hyperphagia in ob/ob mice could increase intake of oxalate itself or oxalate precursors that are converted to oxalate by the liver to increase plasma oxalate and ensuing urine oxalate excretion.

In this study we developed a new LC–MS assay to determine oxalate levels in mouse urine. This assay was more precise than the commonly used colorimetric assay for oxalate measurement. The LC–MS assay showed a positive bias for estimates of oxalate concentration in mouse urine compared to the colorimetric assay. This positive bias was also previously noted with an IC‐HPLC assay that was used for oxalate measurement of urine samples from alanine‐glyoxylate amino transferase (Agxt) gene knockout mice (Salido et al., 2006). We conclude that the colorimetric assay *underestimates* oxalate concentration in mouse urine, although future experiments will be needed to compare the accuracy of LC–MS versus colorimetric assay in mouse urine spiked with known amounts of oxalate. We note that the colorimetric assay has been validated for oxalate measurement in human urine but not mouse urine; the greater variability of the colorimetric assay that we observed in mouse urine could be explained by the ~4‐fold higher total osmolality and ~ 6‐fold higher urea concentration in mouse urine compared to human urine. The higher concentration of solutes in mouse urine may interfere with the colorimetric assay and, combined with the need to modify testing conditions to accommodate the smaller urine volumes from laboratory mice, may contribute to more assay variability.

With regard to urine oxalate excretion in ob/ob mice, our findings differ from Amin et al. (2018) who demonstrated that ob/ob mice excrete higher levels of urine oxalate even when ob/ob and wild type were pair fed with restricted food intake. We propose several reasons for why our findings differ. First, food content and preparation may matter. The diet used in our study (Teklad Global 18% Protein Rodent Diet, TD.2918) was different from the diet used by Amin et al. (Teklad Custom Diet, TD.97184). We also prepared gel food to precisely control the amount of daily food intake. It is possible that food delivery without gel preparation could lead to differences in actual food intake because non‐gel containing food is more apt to crumble and be lost from metabolic cages. Second, the oxalate assay may matter. As stated above, it is possible that the higher level of urine oxalate excretion in wild type mice in our study could reflect the positive bias of our LC–MS assay. Third, the strain and source of control mice may matter. We used wild type C57BL/6J mice as lean control mice, which has been used as a reference for comparing urine oxalate excretion in mouse models of hyperoxaluria (Hatch et al., 2011; Hatch & Freel, 2013; Li et al., 2015; Martin‐Higueras et al., 2016). We acknowledge the possibility that a different control, such as ob/+ mice or wild type mice from other commercial sources, could have accounted for variation in baseline levels of urine oxalate excretion. In this regard, wild type mice in our study excreted more urine oxalate compared to control mice in Amin et al. and other studies (Amin et al., 2018; Hatch et al., 2011; Hatch & Freel, 2013; Li et al., 2015; Martin‐Higueras et al., 2016; Salido et al., 2006). Finally, the way urine oxalate excretion is expressed may matter. We found that ob/ob mice excrete less creatinine into the urine over a 24‐h period compared to wild type mice. Since ob/ob mice weigh more and are obese, the urine creatinine index (24‐h urine creatinine per body weight) was significantly lower in ob/ob mice in all experimental conditions and likely reflects lower lean body mass in ob/ob mice (Almond & Enser, 1984; Bergen et al., 1975; Sáinz et al., 2009; Trostler et al., 1979; Trostler et al., 1982; Warmington et al., 2000). If urine oxalate excretion is normalized to urine creatinine excretion in ob/ob mice, the urine oxalate/creatinine ratio may over‐estimate oxalate excretion in ob/ob mice in a spot or timed urine collection and lead to errors in comparing urine oxalate excretion between ob/ob mice and other mouse strains.

The lack of a role for leptin deficiency or insulin resistance in affecting urine oxalate excretion in ob/ob mice is important for several reasons. First, the primary defect in ob/ob mice is leptin deficiency; leptin signaling might have played a role in regulating urine oxalate homeostasis. When ob/ob mice are administered exogenous leptin, it lowers body weight and improves markers of insulin resistance and systemic inflammation (Frühbeck et al., 2017). Yet in this study leptin replacement at doses that reverse insulin resistance and systemic inflammation does not affect urine oxalate excretion in food‐restricted ob/ob mice. Second, ob/ob mice are also insulin resistant and might have directly contributed to urine oxalate excretion. There have been studies suggesting an association between insulin resistance and hyperoxaluria, and patients with more clinical features of insulin resistance excrete more urine oxalate (Sakhaee et al., 2012). Moreover, when patients with kidney stones become more insulin sensitive, urine oxalate excretion decreases. Stone formers who are given pioglitazone (30 mg/day) for 24 weeks become more sensitive to insulin, have improvements in serum glucose concentration, and excrete less urine oxalate (Maalouf et al., 2019). In our study, reduction of insulin resistance of ob/ob mice by pioglitazone treatment (or leptin replacement) does not lower urine oxalate excretion. These negative findings suggest other mechanisms in obesity underlie the pathogenesis of hyperoxaluria and kidney stone risk.

Recently, Gianmoena et al. (2021) identified alterations in glyoxylate metabolism as a risk factor for hyperoxaluria in mouse models of obesity and human adolescents with obesity and fatty liver. In isolated hepatocytes from ob/ob mice and humans with non‐alcoholic fatty liver disease, there are higher levels of methylation and lower levels of chromatin access in the promoter of alanine‐glyoxylate aminotransferase (Agxt), a key enzyme that converts glyoxylate to oxalate. As a consequence, Agxt expression is reduced so that the affected liver is less able to de‐toxify glyoxylate, and more oxalate accumulates in the liver, plasma, and urine. This defect becomes most apparent when ob/ob mice are fed hydroxyproline, a glyoxylate precursor, resulting in an increase in urine oxalate excretion. It is noteworthy in our study that we did not find evidence of hyperoxaluria in ob/ob mice compared to wild type C57BL/6J mice, although we did not challenge ob/ob mice with hydroxyproline feeding or examine hepatic oxalate deposition. Our findings highlight the complexity of oxalate homeostasis in ob/ob mice and suggest that eating behavior, hepatic glyoxylate metabolism, and intestinal oxalate transport may all conspire to increase urinary oxalate excretion in this mouse model.

One of the most concrete connections between gut microbiota function and human health is illustrated by the role of *O. formigenes* on urine oxalate excretion in kidney stone disease. *Oxalobacter formigenes* is an anaerobic bacterium that degrades oxalate in the gut (Siva et al., 2009) and stimulates intestinal oxalate secretion through yet‐unidentified bioactive factors, ultimately limiting net intestinal absorption and urine oxalate excretion (Arvans et al., 2017). Several clinical studies have linked gut colonization with *O. formigenes* to a lower risk for stone formation in patients with recurrent calcium oxalate stones (Kaufman et al., 2008; Sidhu et al., 1999; Siener et al., 2013). Recent evidence in rodent models suggests that the gut microbiota community affects *O. formigenes* colonization and consequently hyperoxaluria (Miller et al., 2017). However, we found that ablation of intestinal bacteria by antibiotic treatment of ob/ob mice does not affect urine excretion of oxalate or related metabolites, suggesting that gut microbiota does not contribute to hyperoxaluria in this mouse model. The role of gut microbiome in the pathogenesis of hyperoxaluria in patients with kidney stone disease remains to be defined, particularly regarding how gut microbial communities might change with obesity and whether these potential changes in gut microbial communities can directly confer hyperoxaluria.

## 5 CONCLUSIONS

Treatment of ob/ob mice with antibiotics, leptin, or pioglitazone did not change the level of urine oxalate excretion. Ob/ob mice excrete more oxalate into the urine because they eat more food compared to wild type mice. Future work will be needed to standardize the measurement of urine oxalate concentration and to define the range of urine oxalate excretion in wild type mice so that accurate and valid comparisons can be made between wild type mice and ob/ob mice or other mouse models.

### AUTHOR CONTRUBUTIONS

Alan C. Pao and Dylan Dodd designed the study; Alan C. Pao and Dylan Dodd analyzed data; Hong Xiang, Haoqing Chen, Yuanyuan Liu performed the research; Alan C. Pao and Dylan Dodd wrote and revised the manuscript. All authors approved the manuscript.

## ETHICS STATEMENT

This study was approved by the Institutional Animal Care and Use Committees of the Veterans Affairs Palo Alto Health Care System (Palo Alto, CA). All mouse use and welfare adhered to the National Institutes of Health Guide for the Care and Use of Laboratory Animals.

## FUNDING INFORMATION

This work was supported by pilot funding from the Stanford Diabetes Research Center (P30DK116074). A.C.P. was supported by the National Institute of Diabetes and Digestive and Kidney Diseases (DK103758). D.D. was supported by the OHF‐ASN Foundation for Kidney Research (career development award) and by the National Institute of General Medical Sciences (GM142873).

## CONFLICT OF INTEREST

None.

## Supporting information


Figure S1

Table S1

Table S2
