# Supplementary material for: Role of insulin resistance and the gut microbiome on urine oxalate excretion in ob/ob mice
Source: Physiol Rep. 2022 Jul 18;10(14):e15357. doi: 10.14814/phy2.15357 (PMC9294392; doi:10.14814/phy2.15357)
Supplement: Supplementary file 1 — Figure S1 [file PHY2-10-e15357-s003.docx]

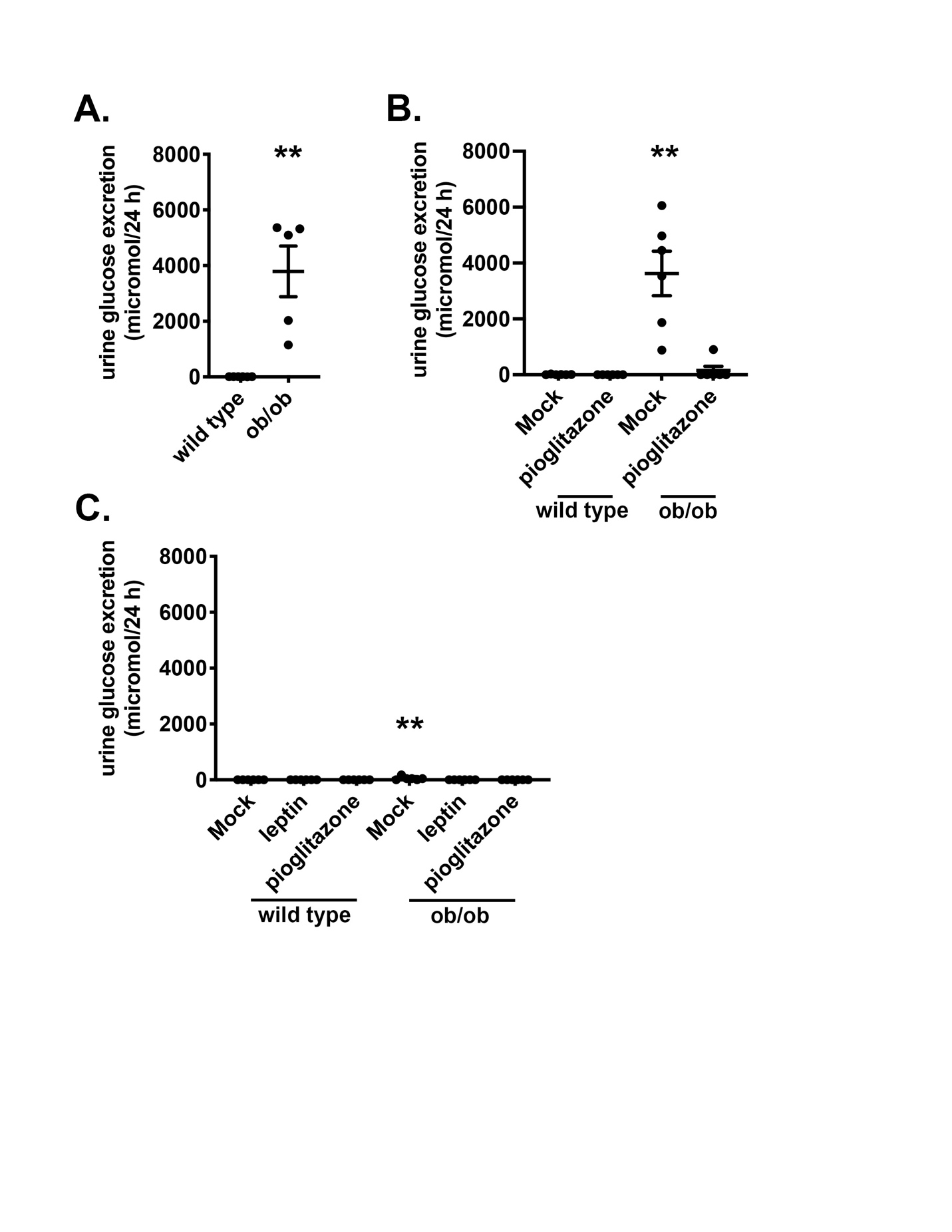


**Supplementary** **Figure 1.** Urine glucose excretion in wild type and ob/ob mice across different experiments. (A) 24-hour urine glucose excretion between wild type and ob/ob mice in experiments with mock treatment. (B) 24-hour urine glucose excretion between wild type and ob/ob mice in experiments with mock or pioglitazone treatment. (C) 24-hour urine glucose excretion between wild type and ob/ob mice in food restriction experiments. ^**^P<0.05 by 2-way ANOVA with Tukey correction for multiple comparisons.
